# Group A Streptococcal Toxic Shock-Like Syndrome in a Male Presenting as Primary Peritonitis: A Case Report and a Review in Japan

**DOI:** 10.1155/2019/4984679

**Published:** 2019-12-17

**Authors:** Shunsuke Sakuraba, Shuhei Ueda, Satoshi Tokuda, Tomoaki Ito, Tomoyuki Kushida, Mutsumi Sakurada, Hiroshi Maekawa, Koichi Sato

**Affiliations:** Department of Surgery, Juntendo Shizuoka Hospital, Shizuoka, Japan

## Abstract

**Background:**

Streptococcal toxic shock-like syndrome (TSLS) is a severe infection caused by group A hemolytic streptococcus. It is clinically characterized by rapidly progressive septic shock and multiple organ failure within just a few hours. TSLS presenting as primary peritonitis is rare, especially in a male. Herein, we report a case of TSLS in a male presenting with primary peritonitis, with a review of 25 cases in Japan.

**Case Presentation:**

A 51-year-old male was referred to our hospital with abdominal pain and hypotension. We made a preoperative diagnosis of peritonitis with septic shock and performed an emergency operation. Intraoperative findings indicated no marked origin of the peritonitis. Preoperative blood culture showed the presence of group A hemolytic streptococcus. The patient required intensive care involving artificial respiration, abdominal drainage and cytokine absorption therapy, and was discharged on postoperative day 25.

**Conclusion:**

TSLS in a male presenting as primary peritonitis is rare. Although this condition is a severe infection, it can be treated by multimodal therapy.

## 1. Introduction

Streptococcal toxic shock-like syndrome (TSLS), also called streptococcal toxic shock syndrome (STSS), was first reported in the mid-1980's [1] and was first described in Japan by Shimizu in 1992 [2]. Although reports of TSLS have become much more frequent as knowledge of this condition has increased, its incidence remains rare, especially in males and the total associated risk of death is 25–40% [3–5]. This severe infection is caused by the production of superantigens derived from streptococcus infection sites and the diagnosis is based on the criteria established by Centers for Disease Control and Prevention in 2011 [6]. Treatment consists of antibiotic therapy and drainage of the source of infection, if possible; however, particularly in cases of multiple organ failure, aggressive fluid management, vasopressor support, ventilation, and renal replacement therapy are often needed. Herein, we report a case of TSLS in a male presenting as primary peritonitis that was managed with multimodal treatment, and provide a review of 25 cases in Japan.

## 2. Case Presentation

**Figure 1:**
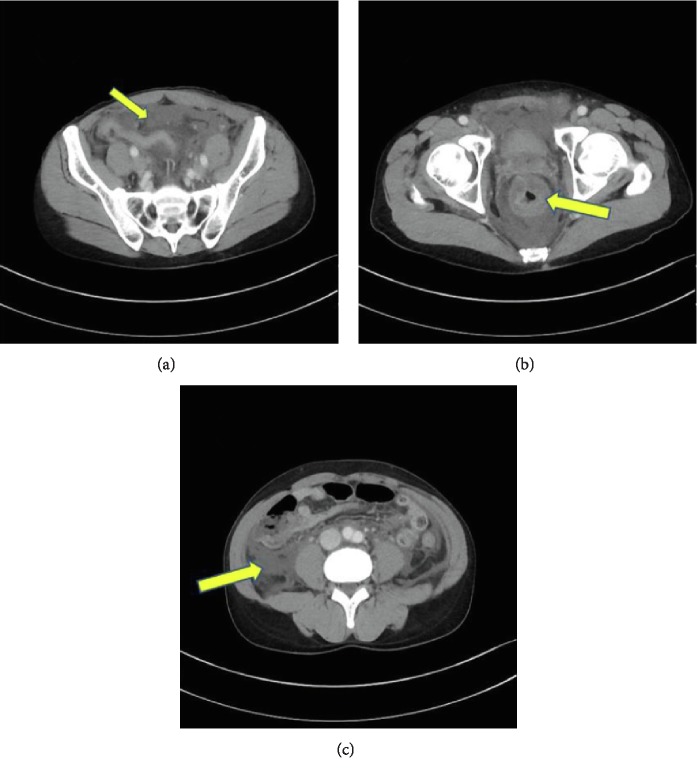
Abdominal computed tomography findings in a patient with primary peritonitis. (a) Computed tomography indicated a small amount of ascites. (b) Computed tomography showed edema of the intestinal membrane. (c) Computed tomography showed edema of the retroperitoneum.

**Figure 2:**
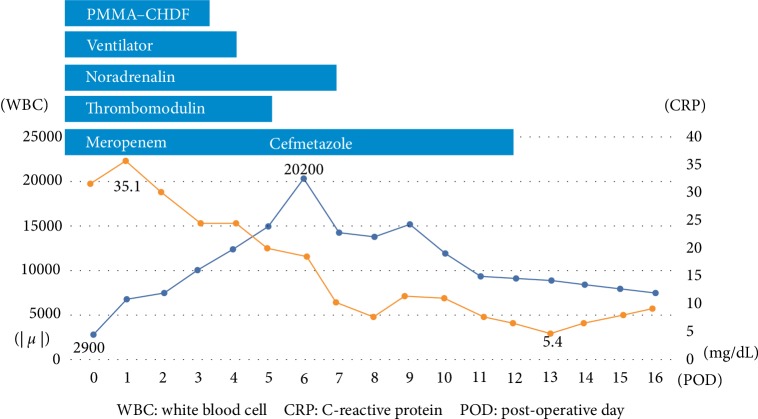
Clinical course of this patient.

A 51-year-old man had abdominal pain 12 hours before seeing a doctor. He was admitted to the previous hospital for the continuous abdominal pain. On the next day, he was referred to our hospital with abdominal pain and hypotension requiring use of an artificial respirator. He had no sore throat and no other symptom before the admission to the hospital. His past medical history was unremarkable, and he was not being treated with any drugs. A physical examination revealed hypotension, with a systolic blood pressure of 70 mmHg, for which noradrenaline 0.18 *µ*g/kg/min was continuously infused. An abdominal examination revealed muscle guarding, rebound tenderness, and an erythematous macular rash over the trunk. A complete blood count revealed no anemia, a white blood cell count of 2.9 × 10^9^/L, and a blood platelet count of 118 × 10^9^/L. Laboratory data showed a C-reactive protein level of 319 mg/L, and a coagulation disorder, prothrombin time ratio: 1.33, fibrin degradation product: 32.5 *µ*g/ml, satisfying acute disseminated intravascular coagulation (DIC) criteria according to the Japanese Association for Acute Medical criteria (JAAM criteria) [7]. Computed tomography indicated a small amount of ascites, edema of the intestinal membrane and retroperitoneum (Figure 1), and no marked gastrointestinal perforation. We initially suspected diffuse peritonitis with septic shock and DIC, and performed an emergency operation. Intraoperative findings revealed a small amount of cloudy ascites, as well as edema of the intestinal membrane and retroperitoneum, but no gastrointestinal tract perforation or necrosis. To rule out retroperitoneum diseases such as a rupture or leak of ureters, ureterography was performed, but no marked origin of the peritonitis was evident. After abdominal cavity irrigation, drains were placed in the pelvic floor and bilateral subphrenic spaces. Ascites looked serous on the next day of the surgery. He was admitted to the intensive care unit and managed with vasopressor support, mechanical ventilation. He was not diagnosed with acute kidney failure, however, to provide treatment for hypercytokinemia, we started continuous hemodiafiltration (CHDF). Since the causative bacteria species was unknown, CHDF using a polymethylmethacrylate (PMMA) membrane hemofilter was performed as cytokine-absorption therapy without the use of polymyxin B-immobilized fiber column direct hemoperfusion (PMX-DHP). The preoperative blood culture showed group A hemolytic streptococcus (GAS). Because of the isolation of GAS, hypotension, coagulopathy (PT ratio, FDP) and erythematous macular rash, TSLS was confirmed. The postoperative blood culture, intraoperative ascites and urine culture were negative, and only the preoperative blood culture, collected at the previous hospital was positive. For antibiotics, carbapenem antibiotic medication was initially administered as empiric therapy, and cephem-based antibiotic was used after confirming drug sensitivity. Forty-eight hours after starting PMMA-CHDF, vasopressors were reduced from 0.2 to 0.07 *µ*g/kg/min and serum lactate acid levels improved from 3.6 mmol/L to 1.4 mmol/L. He successfully recovered from septic shock after the initiation of cytokine-absorption therapy and responded well to the intensive treatment (Figure 2). He was intubated for 5 days and discharged from the hospital on postoperative day 25.

## 3. Discussion

Primary peritonitis is a rare form of peritonitis that results from infectious organisms transmitted through the blood or lymph. Patients with this condition must be distinguished from those with secondary peritonitis, due to gastrointestinal-tract perforation or necrosis, and those with spontaneous bacterial peritonitis [8]. However, because of its rarity, it may be difficult to diagnose this disease prior to surgery. We performed literature searches in Ichushi (Japanese database of medical reports), using the keywords of “Group A hemolytic streptococcus” and “primary peritonitis” from 2000 to 2017. Streptococcal toxic shock-like syndrome (TSLS) presenting as primary peritonitis is rare, [9] and only 25 cases of primary peritonitis caused by group A hemolytic streptococcus have been reported in Japan between 2000 and 2017 (Table 1) [10–31].

According to the literatures in Ichushi (Japanese database), the male to female sex ratio of these patients was 7–18, and the median age at onset was 48 years. As in this case, TSLS presenting as primary peritonitis in a male is very rare, because the ascending infection via the vagina and uterus is thought to be the likely infection route in many female cases. There have been a few cases in which the infection was observed in males. Also, the route of infection was unknown in this case. Almost all patients presented with abdominal pain; other symptoms included fever, malaise, confusion, and the characteristic rash. Our patient exhibited an erythematous macular rash over the trunk, one of the diagnostic criterion of TSLS.

Of the 25 primary peritonitis patients with group A hemolytic streptococcus, 16 (64%) developed TSLS, which suggests that this condition causes septic shock or multiple organ failure at a high rate. However, the mortality rate was 12% (3 of 25 patients), which is relatively low compared with the death rate of TSLS derived from the other organ infection sites. The fact that abdominal drainage of the source of infection and cytokine absorption therapy were performed in many cases may have contributed to the low mortality rate: 23 of 25 patients (92%) underwent abdominal drain placement, and 10 of the 16 patients (62.5%) who developed TSLS received PMX-DHP, PMMA-CHDF, or AN69ST-CHDF. Among the three cases that died, 1 patient was treated without abdominal drainage and 2 received no cytokine absorption therapy; it should be noted that this hypothesis was based on a small number of cases.

Regarding cytokine absorption therapy, 8 of 10 patients (80%) received PMX-DHP, PMMA-CHDF was administered in 2 patients (20%) and AN69ST was administered in one case (10%) (with some overlap). In general, PMX-DHP is used to treat Gram-negative bacterial infections; however, it has recently been recognized that it also acts on Gram-positive infections by absorbing endogenous substances, such as anandamide or 2-arachidonoylglycerol [32–34]. Considering that the underlying mechanism of TSLS is hypercytokinemia induced by superantigens derived from GAS, all cytokine absorption therapies might be useful for TSLS; however, the indications remain to be debated. Among our patients, since the causative bacteria was initially unknown, CHDF using polymethylmethacrylate (PMMA) membrane hemofilter was selected as cytokine-absorption therapy.

The details of the infection route were as follows: 7 patients, via the female genital organs; 4 patients, hematogenous dissemination of pharyngitis; and 14 patients, unknown. This disease is comparatively more common in females since the ascending infection via the vagina and uterus are considered to be a likely infection route. In this Japanese review, among the 18 females, 7 tested positive on a vaginal culture, 6 tested negative, and 5 were unknown.

A combination of antimicrobials with penicillin and clindamycin and intravenous immunoglobulins is recommended for group A hemolytic streptococcus infections [35, 36]. Therefore, these antimicrobials are frequently used in reported cases in Japan. In our case, a definite diagnosis was made six days after surgery based on the preoperative blood culture collected at the previous hospital, so carbapenem were administered as an empiric therapy and we used cephem-based antibiotic secondly according to the drug sensitivity of blood culture.

Although we used thrombomodulin for DIC, there are few Japanese reports of using it. Further studies are needed to confirm the effectiveness of thrombomodulin.

In summary, first, TSLS presenting as primary peritonitis in a male was rare and most of the cases had no prodromal symptoms. Only one male patient had pharyngitis as a precursor. Second, the problem in clinical practice were that there were no characteristic imaging findings or laboratory findings for TSLS with primary peritonitis, and it was difficult to diagnose before treatment. Finally, blood purification therapy would be useful for TSLS, but more cases need to be accumulated.

## 4. Conclusion

We report TSLS in a male presenting as primary peritonitis and review the previous report in Japan. Surgical intervention and cytokine absorption therapy were performed in many cases with TSLS and the most cases recovered. Multimodal therapy involving drainage, cytokine absorption therapy, intensive care and antibiotics may improve the currently high mortality rate.

## Figures and Tables

**Table 1:** Clinical characteristics of the patients.

	Age	Sex	TSLS	Cytokine absorption	Outcome	Infection route	Drainage	Vaginal culture
2000	35	F	No	CHDF	Survive	Pharyngitis	Operation	Negative
2003	54	F	Yes	PMX	Survive	?	Operation	?
2003	38	F	Yes	No	Survive	Vagina	Operation	Positive
2004	67	F	Yes	PMX	Dead	Pharyngitis	Operation	?
2004	40	M	Yes	PMX	Survive	Pharyngitis	Operation	—
2005	38	F	No	?	Survive	Vagina	Operation	Positive
2008	52	M	No	No	Survive	?	Operation	–
2012	63	F	No	No	Survive	?	Operation	?
2012	50	M	Yes	PMX	Survive	?	Operation	—
2012	25	F	No	?	Survive	Vagina	No	Positive
2012	35	F	Yes	PMX	Survive	Vagina	Puncture	Positive
2013	65	F	No	?	Survive	?	Operation	?
2013	70	M	Yes	CHDF	Dead	?	No	—
2013	41	F	Yes	No	Survive	Vagina	Operation	Positive
2014	39	F	Yes	PMX	Survive	?	Operation	?
2015	30	F	Yes	No	Survive	?	Operation	Negative
2015	71	M	Yes	PMX	Survive	?	Operation	—
2015	58	F	Yes	CHDF	Dead	Vagina	Operation	Positive
2016	58	F	Yes	PMMA	Survive	Pharyngitis	Operation	Negative
2016	71	M	Yes	AN69ST, PMX	Survive	?	Operation	—
2017	37	F	No	No	Survive	?	Operation	Negative
2017	42	F	No	No	Survive	?	Operation	Negative
2017	50	F	Yes	No	Survive	Vagina	Operation	Positive
2017	19	F	No	No	Survive	?	Operation	Negative
Our case	51	M	Yes	CHDF	Survive	?	Operation	-

TSLS: Toxic shock like syndrome, CHDF: continuous hemodiafiltration, PMX: polymyxin B-immobilized fiber column direct hemoperfusion, PMMA: PMMA-CHDF, AN69ST: AN69ST-CHDF.
